# Modeling the effector - regulatory T cell cross-regulation reveals the intrinsic character of relapses in Multiple Sclerosis

**DOI:** 10.1186/1752-0509-5-114

**Published:** 2011-07-15

**Authors:** Nieves Vélez de Mendizábal, Jorge Carneiro, Ricard V Solé, Joaquín Goñi, Jean Bragard, Ivan Martinez-Forero, Sara Martinez-Pasamar, Jorge Sepulcre, Javier Torrealdea, Francesca Bagnato, Jordi Garcia-Ojalvo, Pablo Villoslada

**Affiliations:** 1 Division of Neurosciences, CIMA - University of Navarra. Avenida Pio XII 55, 31008 Pamplona, Spain; 2 Computer Science and Artificial Intelligence Department, University of the Basque Country, Paseo de Manuel Lardizábal, 1, 20018 San Sebastian, Spain; 3 Theoretical Immunology Group, Instituto Gulbenkian de Ciencia, Rua da Quinta Grande 6, 2780-156 Oeiras, Portugal; 4 Complex Systems Lab, Universitat Pompeu Fabra, Carrer del Dr. Aiguader, 88, 08003 Barcelona, Spain; 5 Center for Neuroimmunology, Department of Neurosciences. Institute of Biomedical Research August Pi Sunyer (IDIBAPS) - Hospital Clinic of Barcelona. Villarroel 170, 08036 Barcelona, Spain; 6 Neuroimmunology Branch, National Institute of Neurological Diseases and Stroke. 9000 Rockville Pike, Bethesda, MD. USA; 7 Departament de Física i Enginyeria Nuclear, Universitat Politecnica de Catalunya, Rambla Sant Nebridi s/n, 08222 Terrasa, Spain

## Abstract

**Background:**

The relapsing-remitting dynamics is a hallmark of autoimmune diseases such as Multiple Sclerosis (MS). Although current understanding of both cellular and molecular mechanisms involved in the pathogenesis of autoimmune diseases is significant, how their activity generates this prototypical dynamics is not understood yet. In order to gain insight about the mechanisms that drive these relapsing-remitting dynamics, we developed a computational model using such biological knowledge. We hypothesized that the relapsing dynamics in autoimmunity can arise through the failure in the mechanisms controlling cross-regulation between regulatory and effector T cells with the interplay of stochastic events (e.g. failure in central tolerance, activation by pathogens) that are able to trigger the immune system.

**Results:**

The model represents five concepts: central tolerance (T-cell generation by the thymus), T-cell activation, T-cell memory, cross-regulation (negative feedback) between regulatory and effector T-cells and tissue damage. We enriched the model with reversible and irreversible tissue damage, which aims to provide a comprehensible link between autoimmune activity and clinical relapses and active lesions in the magnetic resonances studies in patients with Multiple Sclerosis. Our analysis shows that the weakness in this negative feedback between effector and regulatory T-cells, allows the immune system to generate the characteristic relapsing-remitting dynamics of autoimmune diseases, without the need of additional environmental triggers. The simulations show that the timing at which relapses appear is highly unpredictable. We also introduced targeted perturbations into the model that mimicked immunotherapies that modulate effector and regulatory populations. The effects of such therapies happened to be highly dependent on the timing and/or dose, and on the underlying dynamic of the immune system.

**Conclusion:**

The relapsing dynamic in MS derives from the emergent properties of the immune system operating in a pathological state, a fact that has implications for predicting disease course and developing new therapies for MS.

## Background

**Figure 1:**
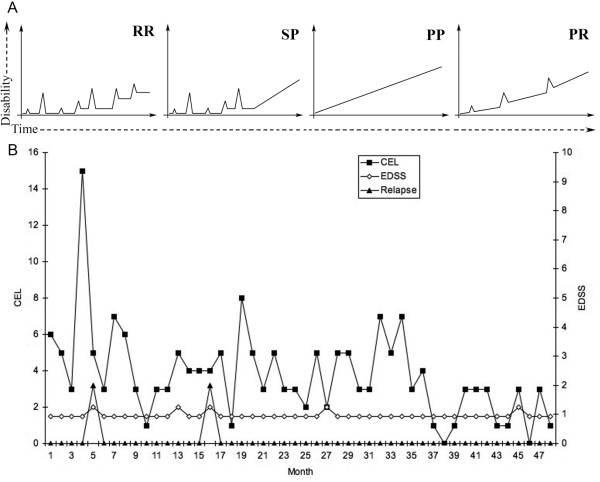
**The relapsing-remitting dynamics of Multiple Sclerosis**. A) Disease subtype classification based in the presence of relapses: relapsing-remitting (RR), secondary-progressive (SP), primary-progressive (PP) and progressive-relapsing (PR); B) Representative patient with MS whom underwent monthly MRI for 48 months. Number of contrast enhancing lesions (CEL; left Y axis), disability (measured with the EDSS scale; right Y axis) and presence of clinical relapses counterpart (no scale)

Multiple Sclerosis (MS) is the prototypic autoimmune disease with relapsing-remitting behaviour [1,2]. Clinical relapses are the defining feature of MS and act as the basis for categorizing different forms of the disease, as a marker to define the disease's natural history and to measure the success of new therapies (Figure 1A). A relapse in MS is a reflection of acute focal inflammatory event in the central nervous system (CNS) that disrupts neural conduction by damaging myelinated axons. It is now known from natural history studies performed using frequent MRI scanning that clinical relapses represent only a small proportion (less than 20%) of CNS inflammatory events, indicated by the presence of contrast enhancing lesions [2-4]. The clinical relapse rate during the relapsing-remitting phase of MS is around one per year and decreases as the disease advances [5,6]. In MS, clinical relapses generally last for a month with spontaneous partial or full recovery afterwards. Their distributions along time have not been associated with any specific pattern or precipitator [2,7] although it has been estimated that the presences of such relapses are preceded in one third of cases by either infections or stressful events [8,9]. In any case, a clear understanding of environmental factors driving the presence of relapses as well as the cellular and molecular mechanisms governing the relapse onset and resolution is still lacking.

Although pathogenesis of MS is still unknown, it is proposed as a T-cell-mediated autoimmune disease, since its characteristic activity pattern, including relapses, is related to the temporally and spatially segregated effects of activated T cells [10,11]. Healthy animals and humans contain auto-reactive effector T cells in the peripheral repertoire [12]. Although the activity and function of auto-reactive T cells in humans is not well understood, studies in non-human primates have shown that these cells can be activated, and may occasionally trigger a T-cell-mediated attack against the CNS [13]. However, the activation and clonal expansion of these effector T cells (Teff) is believed to be inhibited by peripheral tolerance mechanisms, among other factors by the presence of active regulatory T cells (Treg) [14-18]. As a matter of fact, deficiency in Treg results in a fatal autoimmune syndrome affecting multiple organs in mice [19,20] and humans [21,22]. Moreover, several reports suggest MS and type 1 diabetic patients may have an impaired Treg function and an imbalance in Teff-Treg homeostasis [23-25]. On the other hand therapeutic CTLA-4 blocking with monoclonal antibodies (ipilimumab) in cancer patients induces autoimmunity [26]. In the periphery, for a given age and genetic background, the Treg population represents a stable proportion of the CD4^+ ^T cells in the steady state (10%), suggesting that the homeostasis of Teff and Treg are tightly co-regulated [27]. Thus, homeostasis of Treg is likely to be an important process in the proper functioning of the immune system as well for controlling self-reacting Teff cells and preventing autoimmunity [20,28,29].

In order to gain insights in the cellular events leading to the relapsing dynamics, we develop a computational model of the adaptive immune system. We hypothesize that the cross-regulation between Teff and Treg cells that works as a negative feedback, coupled with stochastic processes such as common infections (noise), is able to buffer oscillations in the functioning of the immune system, allowing at the same time to create an immune response when it is required [30]. However, under certain circumstances, such as in autoimmune diseases, the system is able to create a stable oscillatory behaviour that allows the activation and expansion of self-reacting Teff cells. Activation of Teff will induce tissue damage, the counterpart of clinical relapse. In this study we attempt to explain what conditions are necessary for triggering the relapsing-remitting autoimmune response in MS and why in most cases it is not a chronic-progressive process from the beginning. Given the key role of relapses in the study and management of MS and other autoimmune diseases, a broad understanding of relapsing dynamics is important to promote accurate diagnosis, patient management and treatment decisions.

## Results

### Modelling the adaptive immune system reveals the cross-regulation between Teff-Treg populations a control mechanism that maintains immune tolerance

**Figure 2:**
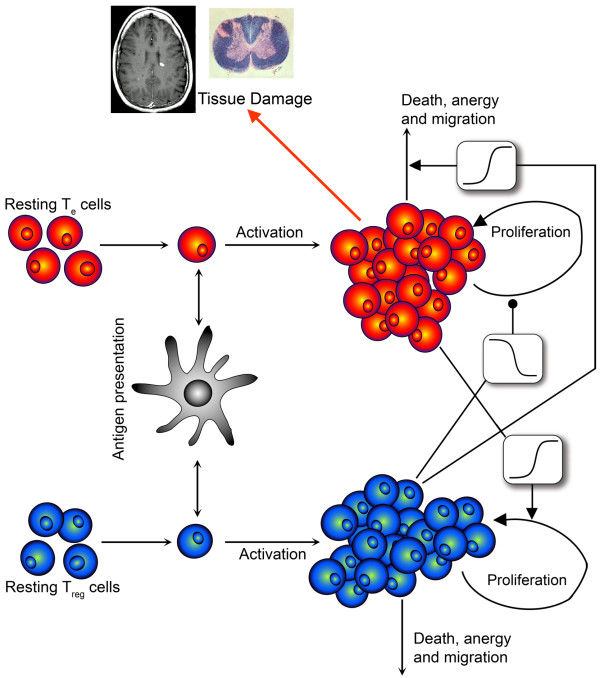
**Model of the adaptive immune system**. The model of the adaptive immune system is comprised of four parts: 1) the generation of Teff and Treg cells from thymus; 2) T-cell activation by APCs; 3) the cross-regulation modeled by the Teff-Treg loop; and 4) T cell activation and memory populations in the tissue. The Teff-Treg loop is composed of a negative feedback between the two populations (Teff in red and Treg in blue). The model can be found in the additional files, and the parameters and initial conditions are listed in Table 1.

**Figure 3:**
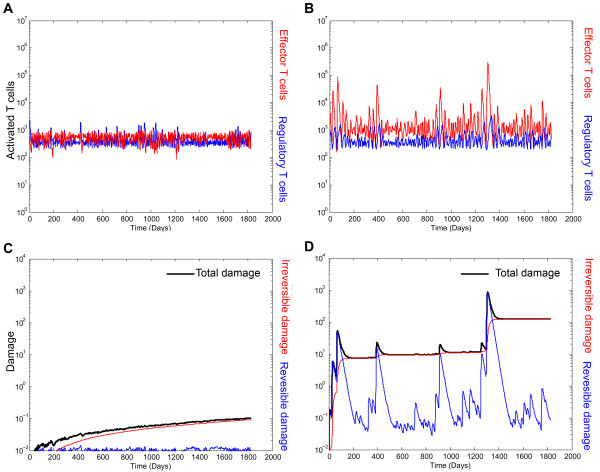
**Simulations of the computational model in healthy and autoimmune state**. Plots show representative simulations of the antigen-specific activated-Teff (red) and Treg (blue) population in log scale over 5 years. By changing the parameters of the Teff-Treg loop, we were able to reproduce two dynamics:A)Healthy state in which the Teff and Treg populations fluctuate at low levels indicating that the system is not generating an immune response, nor immunopathology;B) Autoimmune dynamics due to a failure in the cross-regulation (Teff-Treg loop), provoking the expansion and contraction of activated Teff cells. C, D) Simulations of tissue damage: reversible damage (blue), irreversible damage (red) and total damage (black): C) In healthy configuration the functioning of the immune system (blue line) do not induce significant tissue damage (black line); D) in autoimmune configuration the peaks of activated Teff induce tissue dysfunction and damage (red line), leading to the relapsing dynamics (back line).

We aimed to probe by means of a mathematical model (see methods and additional files 1, 2 and 3, Figure 2) whether the relapsing-remitting dynamics in MS can emerge as a result of the intrinsic control properties of the immune system that allow it to oscillate (e.g. the Teff-Treg loop) [30]. We observed two different kinds of dynamics: healthy regime and autoimmune regime (Figure 3). Working under a healthy configuration, activated Teff-Treg populations remain in a dynamic (oscillatory) balance without producing immune responses (Figure 3A), the equivalent of immune homeostasis. Such activated Teff dynamics does not produce tissue damage (Figure 3C). However, by decreasing the maximum activation and proliferation rate of Treg (*α_R_*) and without changing the production or activation rate of self-reacting resting T-cells, immune homeostasis was lost and spontaneous immune responses emerged in the absence of infectious agents, mimicking the relapsing dynamics of autoimmune diseases (Figure 3B). The presence of activated Teff cell peaks produces both reversible and irreversible damage. The sum of both represents the clinical relapses equivalent to the one observed in MS patients (Figure 3D). The presence of the Teff-Treg loop restores the homeostatic levels providing an explanation for why relapses recover and the disease does not become chronic-progressive.

A significant breakdown of central immune tolerance might be expected to produce an autoimmune phenotype, since the frequency of self-reactive lymphocytes would be increased, as occurs in the IPEX or APECED syndromes that are caused by mutations in the Foxp3 or AIRE genes, respectively [22]. However, performing a sensitivity analysis by increasing the generation of self-reactive T cells, we found that if the Teff-Treg loop parameters are maintained within homeostatic values (healthy regime), this module can cope with such increase of self-reacting Teff, even if the amount (perturbation intensity) is drastically augmented (data not shown). Thus, immune tolerance is maintained and the appearance of autoimmunity is prevented. In order to observe the induction of autoimmune diseases in this context, it was necessary to tune down the Teff-Treg regulatory loop. Therefore our model indicates that genetic mutations affecting central tolerance are not sufficient to develop autoimmune diseases but require an impaired Teff-Treg cross-regulation. This is in agreement with the experimental finding that Treg function is impaired in such hereditary autoimmune diseases [31]. Finally, we observed in our simulations, using same parameters but different seeds, that the distribution of activated Teff cells peaks (relapses) are produced at different intensities and moments, indicating spontaneous heterogeneity in the temporal distribution of relapses (Additional File 4, Figure S1). Such differences account for a different behavior of the reversible and irreversible damage, indicating clinical heterogeneity.

### Decreasing the strength of the Teff-Treg cross-regulation favours autoimmunity

We explored the autoimmune dynamics in the parameter space that controls the Teff-Treg regulatory loop, repeating the procedure with different input seeds (see methods) in order to reproduce the stochasticity in the activation of the immune system by pathogens. Although other parameters can influence in the outcome dynamic (i.e. *h, ke, kr*...), it was *α_R _*which provokes the characteristic relapsing-remitting peaks. As we will see *α_E _*turned out to be an important parameter as well. Because of that we performed a sensitivity analysis of both parameters running simulations over 1,825 days (time step of 0.05 days) for each pair of proliferation rate values in the range of *α_E _*= [1:2] and *α_R _*= [0.25: 1], with 0.05 as step value (uniform intervals) (Additional File 5, Figure S2). Since we are dealing with stochastic differential equations, 200 different realizations were performed for each pair of values of the scanned parameters. Peaks of activated Teff cells were only observed when there was a reduction in the strength of the Teff-Treg regulatory loop, and mainly when the Treg response was slower (Additional File 5, Figure S2A). We observed that the simulations only produced high peaks of Teff activity in a small region of the parameter space. As expected, the highest peaks of activated Teff cells were observed when Treg activity and proliferation was minimal. However, the highest peaks did not always occur when Teff proliferation rate was at its highest. A decrease in the Teff proliferation rate for a given seed does not necessarily imply less severe relapses and, as a matter of fact, it could produce an increase in relapse intensity. Such apparently counterintuitive consequences are due to the strong interaction between the time dynamics of the system and the time at which the noise input occurs, as we will see further on. For average results (Additional File 5, Figure S2B), the population median tends to grow as the maximum Teff proliferation rate increases, as well as when the maximum Treg proliferation rate decreases.

**Figure 4:**
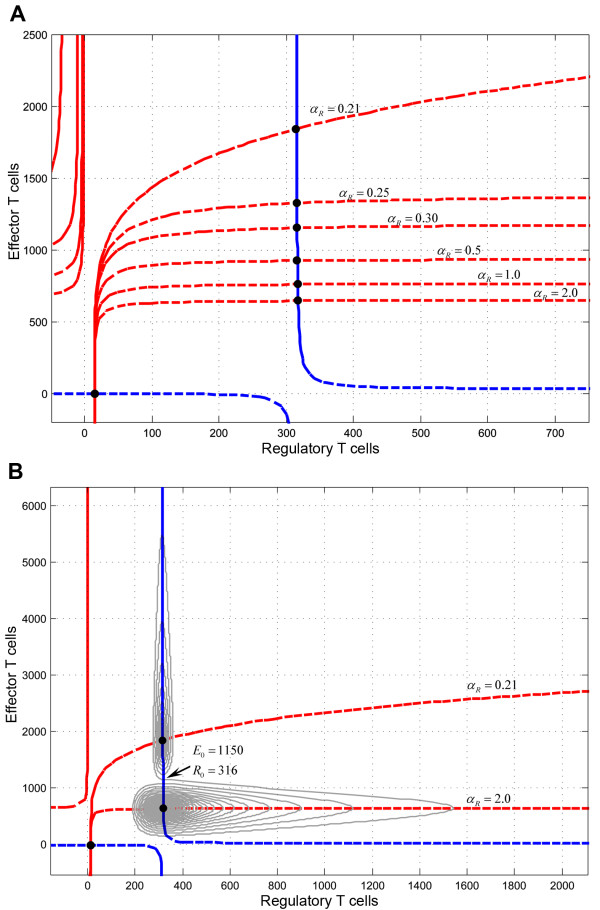
**State-space analysis of the Teff-Treg loop**. The state-space analysis was done with the simplified T-cell population model (eq. 2). The X axis represents the activated Treg population and the Y axis activated Teff cells. A)By plotting both populations with different levels of α_R _the equilibrium point moves to higher numbers of Teff cells, remaining practically the number of Treg cells in the same point.B)State-space plot corresponding to the two extreme values for α_R_: 0.21 and 2.0, showing an abrupt change in the dynamics.

The previous model can be further simplified in order to keep the dynamically relevant compartments influencing the time behavior. In the reduced model, the T-cell population system is represented by just two variables, namely E and R, representing the size of activated T cell populations and the stochastic effects were eliminated substituting *R_E_, R_R _*respectively by their expected values *Λ_E_, Λ_R _*(see eq. 5-6 in methods). In absence of stochastic events, the system has a stable point (Figure 4). This point is an attractive point and the system oscillates with decreasing amplitude until reaching the equilibrium point (Figure 4B). The value of Teff and Treg cells reached in the equilibrium depends on the specific values of every parameter of the model and does not depend on the initial conditions of the levels of activated Teff and Treg cells. We observed that when *α_R _*decreases, the equilibrium point moves to higher numbers of Teff cells, while Treg cells remain practically the same point (Figure 4A). Moreover as *α_R _*decreases the shape of the spirals gets longer along the Y axis, corresponding to Teff cells, and becomes radically narrower in the X axis, which corresponds to Treg cells (Figure 4B). The sensitivity analysis of the system showed that the shape of the spirals do not significantly change when the maximum Teff proliferation rate *α_E _*changes, although the value of the equilibrium point increases in the Treg axis. If we carry the system to extreme values, *α_R  _*> 1, this latter dynamic might represent a hypersensitive Treg response, which could be beneficial to prevent autoimmunity, although perhaps at the cost of not being able to control infections or even, inducing immunodeficiency. This mechanism could speed up the Treg response, allowing a pathogen to establish a chronic infection. As an example, it has been proposed that many pathogens responsible for persistent chronic infections highjack immunological regulatory mechanisms [32]. In summary, our analysis suggests that the Teff-Treg regulatory loop is critical for the outcome of the system (tolerance, response to infections, autoimmunity and immunodeficiency).

### Predicting relapses in MS

**Figure 5:**
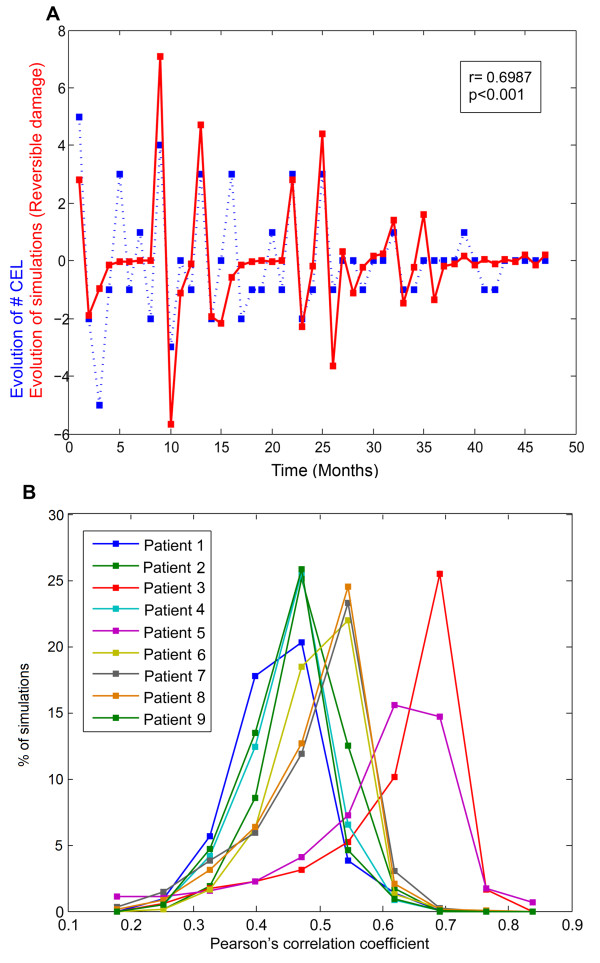
**Prediction of relapses for clinical datasets **A) Simulations of CEL time series: simulations of the reversible damage in months (in blue) from the computational model reproduce the dynamics of CEL dataset from patients with MS (in red); B) Pearson's correlation coefficients between CEL series and reversible damage. Each correlation distribution was obtained by comparing 2,000 simulated series and each patient series.

In order to evaluate and validate our model, we compared computational simulations of the reversible damage (which are highly correlated to the T cell infiltration in the CNS) with the contrast enhancing lesion (CEL) time series from 9 patients with MS. The model was able to partially forecast the CEL time series (Figure 5A), with correlation distributions centered on coefficient values of 0.55 and in some cases up to 0.75 (Figure 5B). These results indicate that Teff-Treg cross-regulation is a key element in governing the oscillatory behavior of the immune system and drive the presence of relapses in MS, but at the same time indicate a significant component of stochasticity in the generation of clinical relapses, limiting our predictive ability.

### Dynamics of the Teff-Treg regulatory loop after perturbation: Implications for immunotherapy

**Figure 6:**
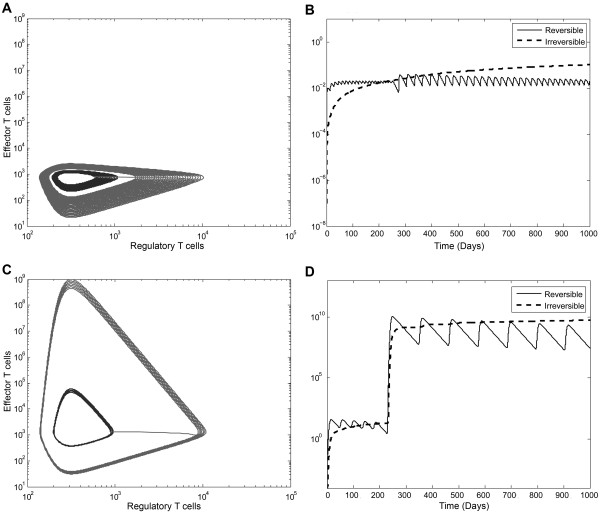
**Modeling Treg cell therapy**. A)Healthy configurations: while the system is moving clockwise on a spiral trajectory that moves towards the equilibrium point, the model was perturbed with an impulse of Treg cells (only a portion of the trajectory is shown for clarity). The perturbation was introduced near to the maximum possible value of activated Treg. After the impulse, the system jumps to a more distant spiral, where the new maximum value reached by the activated Teff is greater than that on the initial trajectory. B)In the healthy configuration reversible and irreversible damage result almost imperceptible. C) Autoimmune configuration: while the Teff-Treg loop is driving an autoimmune regime, the model was perturbed with the same impulse as above. The effect of the perturbation was qualitative similar to that in the healthy regime, but not quantitatively, showing the hypersensitivity of this kind of regime in the model. D)Both reversible and irreversible damage resulted evident when the system was working under autoimmune conditions.

The effects of Treg and Teff perturbations on the system were studied in order to gain insight into the dynamics of the immune system, mainly on the effect of noise, as well as to predict the effect of immunotherapies targeting either effector or regulatory T cell populations. By analyzing the *E*-*R *state-space before and after the perturbation, we identified the different outcomes in the system. In the absence of perturbations, the trajectory of the state-space of both populations is a spiral that moves clockwise until reaching the equilibrium point, and the change of the initial conditions produces a new trajectory that also moved clockwise towards the same equilibrium point (see Figure 6: only a portion of the trajectory is shown for clarity). The effect of introducing an impulse of either naïve Teff or Treg cells produced a jump to another trajectory. This is equivalent to restarting the model under different initial conditions, the equilibrium point remaining the same.

From the therapeutic point of view, modulating the levels of Teff and Treg populations as possible immunotherapies has several implications that a priori cannot be intuitive. The time at which the therapy is introduced may be important, since depending on this factor the effect can be beneficial or deleterious (Additional File 6, Figure S3). Thus, if a therapy increasing the levels or activity of Treg is administered when the dynamic of the effector population is decreasing, the effect of the Treg immunotherapy would be deleterious. In the same way, although an immunotherapy with Teff cells would be deleterious for autoimmunity, depending on the dose timing could be protective. If a dose with Teff cells is administered when the dynamic of the regulatory population is decreasing, the effect of the Teff immunotherapy would be beneficial although it can seem paradoxical. This prediction can explain why targeting IL-17A, an interleukin with pro-inflammatory functions, might not have beneficial effects if they are administered at the wrong time or dosage [33].

For example, for modeling cell therapy with Treg cells, we compared the sensitivity to a Treg perturbation under health (*α_R _*= 1) and autoimmunity (*α_R _*= 0.25) using the same conditions by analyzing the changes in the trajectory of the phase-space between Teff and Treg cell populations after Treg perturbation and the effect in the dynamics of reversible an irreversible damage (Figure 6). Under healthy configurations and after the perturbation (Treg therapy), the system jumps to a new trajectory (Figure 6A) where the new maximum value reached by activated Teff is higher than in the initial trajectory. However, in the autoimmune condition and after the Treg impulse, the system also jumps to a new more distant trajectory (Figure 6C). The model was more sensitive in this autoimmune regime and while the maximum value of activated Teff cells was less than 10^4 ^cells in the healthy regime, it reached more than 10^9 ^activated Teff cells in the autoimmune regime. In consequence, the damage both reversible and irreversible produced is very noticeable during an autoimmune regime (Figure 6D) while it remains insignificant during a healthy regime (Figure 6B).

## Discussion

The study of the relapsing-remitting dynamics of MS and other autoimmune diseases can have practical benefits for patient management. The unpredictability of relapses in MS is one of the most disturbing aspects of the diseases reported by patients. Given the key role of relapses in the management of the disease, a broad understanding of relapsing dynamics is important to perform an accurate prognosis, improving patient's management and therapeutic decisions. In order to gain insights in the mechanistic basis of this relapsing dynamics we developed a mathematical model of the adaptive immune system. We aimed to assess the hypothesis that the characteristic relapsing-remitting dynamics of autoimmune disease emerges as a result of the intrinsic control properties of the immune system. Based on our simulations, we found that immune tolerance, defined in our model as the capacity to cope the activation of effectors T cells, is an emergent property of the Teff-Treg cross-regulation. This indicates that the Teff-Treg loop is a powerful control module that regulates the adaptive immune system when activated by stochastic environmental factors. A pathological dynamic regime of the Teff-Treg loop created a pulsing dynamics in which the expansion of the Teff population transiently escapes the control of Treg population, creating the relapses typical of autoimmune diseases such as MS. In our model, relapses mainly arise upon the failure in the Treg response and were mainly driven by stochastic process that might correspond either to thymic production of new self-reacting T cells or from random sporadic infections. Interestingly, the frequency of such stochastic events where not the main factor producing relapses, but the severity in the dysfunction of the Teff-Treg cross-regulation was the main responsible of relapse frequency and severity. This finding can explain why the relapse activity in patients with MS is quite stable during the relapsing-remitting phase, because it would mainly depend on the dysfunction of the immune system, but make relapses very difficult to predict.

Our model shows that the pathologic dynamic regime in autoimmune conditions become stationary and makes the autoimmune process chronic but relapsing instead of progressive. In this scenario, autoimmunity can be considered as a dynamic disease [34,35], in which the pathological state arises through the emergence of stationary stochastic dynamics in the immune system that overcomes immune homeostasis. Although our study was mainly inspired in the dynamics of MS, we believe it can provide insights about the dynamic of other autoimmune diseases, since many of them also have relapses (e.g. Lupus or Rheumatoid Arthritis) but also chronic, non-relapsing autoimmune diseases such as type 1 diabetes might also display relapsing inflammatory bursts [36]. Thus, our study supports the view of autoimmune diseases as complex disease produced not by a single molecular or cellular event or governed by environmental challenges, but rather by the combination of many factors that deregulate the control mechanisms in the immune system [37].

Our study highlights the critical role of the cross-regulation of T cell populations in peripheral tolerance and in the generation of autoimmune diseases. Although both the loss of the central tolerance and the impairment of the Teff-Treg loop can contribute to the generation of autoimmunity, our model suggests that the breakdown of cross-regulation and not central tolerance leads to this kind of relapsing autoimmune dynamics. This result is in agreement with the fact that the majority of autoimmune diseases are sporadic and not related with mutations in genes controlling central tolerance, such as in the IPEX or APECED syndromes [22], and that impairment of Teff-Treg population is also required even in monogenic autoimmune diseases [32]. Another conclusion from our study is that the autoimmune diseases can result from the weakening of the peripheral tolerance, particularly the control exerted by Treg over effector/auto-reactive T cells. There is already experimental evidence supporting a role of such control loop on the prevention of some autoimmune disease. Particularly there is evidence of several genetics defects weakening this loop that are clearly associated with autoimmunity [22] as well as previous mathematical models supporting this idea [38]. Our results show that defects on central tolerance (particularly those increasing the frequency of generation of auto-reactive T cells) might not be sufficient to induce autoimmunity. There is already experimental evidence proving this fact in animal models. Particularly, transgenic mouse model have shown that a thymus generating more than 90% of its total output of a single anti-MBP auto-reactive T cell clones without causing autoimmunity, because of the peripheral control exerted by the remaining 10% of the T cell repertoire that happens to contain Treg [39].

Previous models of cross-regulation between effector and regulatory cells have shown bi-stable regimes [29,40]. In one of the stable points, corresponding to the healthy states, the Treg population controls the effector population, while in the second stable point, interpreted as the autoimmune state, effector cells outcompete or predominate over regulatory cells. These models provide an explanation for the etiology and natural history of chronic-progressive autoimmune diseases such as type I Diabetes or the progressive subtypes (non-relapsing) of Lupus or MS, as the result of a switch to the pathology stable attractor. However, relapsing-remitting dynamics of autoimmune diseases such as those found typically in MS, Rheumatoid Arthritis or Lupus still lack of a theoretical framework. The major contribution of the model introduced in this paper is a dynamical explanation for such relapsing-remitting dynamics. Even though the cross-regulation makes that both populations always stay within the basin of attraction of a unique steady stable point, there are configurations whose trajectories produce temporal relapses in presence of environmental stochastic events. One basic assumption of our model is that the oscillatory dynamics of the immune systems will depend the prey-predator model with stochasticity [41]. However, in our model the stochastic environmental factors were modeled as a train of impulses influencing the Teff growth (prey) rate and the Treg death rate (predator). For this reason, our model differs from previous predator-prey models because the stochasticity component come from introducing either Teff or Treg randomly using a train of impulses, independently of the T cell population density used in other models [41].

Our results also show that the onset of the relapse is triggered by stochastic events such as the random thymic generation of auto-reactive Teff or Treg cells or sporadic common infections that activate the immune system. However, the duration of each relapse and the overall relapsing frequency are under the control of the dynamics of Teff-Treg loop. This result might help to explain why previous attempts to identify external factors triggering relapses failed to provide clear explanations [2,11]. Moreover, predicting the appearance of new relapses will be difficult, not only because we would lack sufficient knowledge about the dynamics of the immune system in a given individual at a given time, but also due to the influence of the stochastic events.

In our study we model the reversible tissue damage in order to provide a comprehensible link to the active lesions in CNS observed in patients with MS. Therefore, we compared long-term simulations of reversible tissue damage with the CEL dataset from patients with MS. The simulations obtained closely reproduced the oscillatory behavior of the CEL dataset, obtaining moderate to high correlations and reproduced the two-phase behavior (the relapsing-remitting dynamics). In this way, this model explains dynamics of autoimmunity with a basic cyclic nature. This is important, since several human autoimmune diseases are documented to have a cyclic behavior, although this is not the only class of dynamics observed for autoimmunity (e.g. type 1 diabetes). Moreover, mathematical models of T cell vaccination have explored the dynamics of the immune system after generating anti-idiotipic Treg for switching-off the autoimmune response, explaining the cyclic dynamics of autoimmune disease [42,43]. However, the limited predictive ability suggest that either other biological factors not include in the model or, more probably, the stochasticity of T-cell activation due to random infections might account for a significant proportion of the temporal distribution of relapses. Also, although our model is able to reproduce the relapsing dynamics of MS, other models considering other factors might also be able to provide similar explanations.

From the therapeutic point of view, our results may have several implications. First, our analysis indicates that autoimmunity is a dynamic phenomenon. Thus, perturbing the Teff and Treg populations might produce different outcomes depending on the control parameters of the immune system in a given patient and the timing of the intervention, but without modifying the underlying dynamics. Accordingly, therapeutic approaches to treat autoimmune diseases that involve either decreasing Teff populations (e.g. through chemotherapy, anti-CD52 or anti-CD20 mAb therapy) or increasing Treg populations (e.g. Treg cell therapy) will not cure the disease, since they are aimed to keep the values of both populations in the range observed in healthy state but without restoring the control of the immune system to that of the healthy state. From a systems biology perspective, therapeutic interventions should be designed to restore the dynamics of the system to the healthy state or at least to a less deleterious dynamic [44,45]. In order to efficiently modulate the dynamics of the immune system it is necessary to know in which region of parameter space the immune system of a given patient is acting at any time, as well as to identify which control mechanism can be targeted [44,46]. As we found in the perturbation analysis, considering the outcome of increasing the Treg population or decreasing the Teff population to treat autoimmune diseases, intervening at different times and with different perturbations, might be beneficial. The timing and dose will be specific for a given patient or subgroup of patients, implying the need for personalized medicine. Nevertheless, it will be necessary to translate the critical parameters to specific molecular and cellular markers of the immune system in order to be able to apply it to human immunotherapy [44,45].

Our study has several limitations. As commented before, despite the emerging importance of Treg in the immune system, fundamental parameters of the biology and homeostasis of these cells, such as their lifespan, turnover, and recirculation properties remain unknown. Also, while we analyze the cross-regulation of Teff and Treg populations from a systemic point of view, many other aspects of the immune response that may also be important, were not contemplated, such as the innate immune systems activity, regulation of the effector response in the tissue, the role of the T-cell immune repertoire or the control of the immune response on T-cell activation [37,47]. However even with this simplified model of the immune system, we were able to show that autoimmune diseases can arise as a dynamic phenomenon and we could identify the critical contribution of the Teff-Treg loop in the control of the immune response, providing a theoretical framework for the understanding of the relapsing dynamics in autoimmunity. Also, the induction of an autoimmune response in our model requires some minimal changes in the parameter values. This may represent the accumulation of the genetic background (e.g. HLA class II susceptibility alleles (DRB1*1501), IL2R or IL7R for MS) [48], in conjunction with the shaping of the immune repertoire during ontogeny and the presence of stochastic infectious challenges for generating individuals susceptible to develop autoimmune diseases.

## Conclusions

The relapsing dynamic in MS may derive from the emergent properties of the immune system running in a pathological state, a fact that has implications for predicting disease course and developing new therapies for MS. This pathological state might be produced by the combination of many factors that deregulate the control mechanisms in the immune system (instead of by a single molecular or cellular event). Due to the fact that the results of this study are qualitative rather than quantitative, the conclusions must be considered as highly plausible conjectures, which have to be experimentally tested.

## Methods

### Subjects

Data for this study were previously generated at the NINDS-NIH [49]. Patients whose data are presented were enrolled in a NIH/NINDS/IRB approved protocol. Each subject has signed an informed consent prior to participate to the study. We studied 9 patients with RRMS, free of immunomodulatory treatment, who underwent monthly MRI with gadolinium for 48 months. We recorded the number of CELs for each consecutive month on the MRI, the Expanded Disability Status Scale (EDSS) for measuring clinical disability and the presence of clinical relapses (see additional files). Patients were recruited after obtaining wrote informed consent. The study was approved by the IRB at NIH.

### Mathematical modeling

The model was simulated in the VENSIM (Ventana Systems, Inc., MA, US) and MATLAB (The Mathworks, MA, US) computing environments. The initial values, parameters and their references are shown in Table 1. Despite the emerging importance of Teff and Treg in the immune system, fundamental parameters of the biology and homeostasis of these cells, such as their lifespan, turnover, and recirculation properties remain unknown. The parameters were estimated using goodness of fit plots.

**Table 1:** Parameters of the model

Intial Conditions	Symbol	Values
Resting Teff cell population size	*E_r_*	E_n0 _= 0 cells
Resting Treg cell population size	*R_r_*	R_n0 _= 0 cells
Activated Teff cell population size	*E*	E_a0 _= 1000 cells
Activated Treg cell population size	*R*	R_a0 _= 200 cells
Reversible Damage	*l*	*l_0 _= 0*
Irreversible Damage	*L*	*L_0 _= 0*

**Parameter description**	**Symbol**	**Values**

Antigen Presentation	*δ*	1 day^-1 ^(35)
Anergy	*β*	0.01 day^-1^
Memory	*η*	0.01 day^-1^
Maximum Teff proliferation rate	*α_E_*	[1:2] day^-1 ^(36)
Maximum Treg proliferation and activation rate	*α_R_*	[0.25:2] day^-1^
Teff death, anergy and migration Rate	*γ_E_*	0.2 day^-1 ^(37)
Treg death, anergy and migration Rate	*γ_R_*	0.2 day^-1^
Teff cell population sizes leading to half maximal effect counterpart	*Ke*	1000 cells
Treg cell population sizes leading to half maximal effect	*Kr*	200 cells
Hill coefficient	*h*	5
Reversible damage rate	*d_1_*	1 day^-1^
Irreversible damage rate	*d_2_*	0.02 day^-1^
Threshold	*a*	22800 cells
Recovery	*r*	0.1 day^-1^

#### The T-cell population dynamic

The model is a simplification of the adaptive immune system focusing on the activation of T lymphocytes, in their proliferation and subsequent migration to tissues where they exert their effects. The T-cell population variables represent pool of antigen-specific T cells participating in a given immune response and not the overall T cell repertoire. The conceptual core used to develop this model (Figure 2) basically has three parts: Teff-Treg regulatory loop, the stochastic processes and the tissue damage production.(1)(2)(3)(4)

where *E_r_, R_r_, E, R *represent the size of the resting Teff, resting Treg, active Teff, active Treg cell population respectively. Several parameters are assumed to be identical for both Teff and Treg types: η is the rate at which the cells return to the resting state; δ is the cell activation rate; β is the rate at which the resting cells become anergic or die. Other parameters were assumed to be cell type specific: *I_E_, I_R _*are the stochastic inputs of Teff and Treg cells respectively; *α_E_, α_R _*are maximal proliferation rates; *γ_E_, γ_R _*the clearance rates. The cross-regulatory interactions between the Teff and Treg populations are modeled as Hill functions, where *k_E_, k_R _*are respectively the Teff and Treg population densities/sizes leading to half maximal effect on their counterpart. Finally, *h *is the Hill coefficient, which controls the response sensitivity. Table 1 contains the list of parameters and values of the model.

In order to solve the model analytically, the T-cell population system was reduced to the two variables *E *and *R *representing the size of activated T cell populations. To eliminate the stochastic effects, the terms representing the influx from resting cell populations were set to constant substituting *R_E_, R_R _*respectively by their expected values Λ_E_, Λ_R _respectively.(5)(6)

#### Tissue damage

We coupled to the T-cell population model (eqs. 1-4, Figure 3A) with two additional equations that represent the damage caused by activated Teff cells on the target tissue. With such equations we aim to provide a comprehensible link between autoimmune activity and clinical relapses in patients with MS:(7)(8)

where *l, L *are reversible and irreversible damage respectively; d_1_, d_2 _are the rate constants at which the damage is produced and recovered respectively; and *E' = (E/a)^2 ^*is the second order effect of Teff cells on damage. *l *and *L *are output variables that are affected by, but have no feedback on the T cell population dynamics.

### Theory

The model was designed according to certain assumptions. Such assumptions qualitatively describe relationships that have been experimentally observed.

#### The Teff-Treg regulatory loop

Teff-Treg regulatory loop was modelled as a negative feedback loop between two competing populations, activated Teff and activated Treg cells. This negative feedback is the consequence of a negative influence of Treg over Teff, and a positive influence of Teff over Treg [14-18]. It is essentially a prey-predator system, where Teff cells act as prey and Treg cells as predators.

Treg inhibition of Teff in the model accounts for different known mechanisms: suppression by competition for growth of survival factors as IL-2 [16,18], cell-to-cell contact inhibition [16], secretion of immunosuppressive cytokines and the induction of apoptosis both in vitro and in vivo [50]. In this model, the Treg population has a negative effect on Teff proliferation. There is an inhibition of Teff proliferation due to the presence of activated Treg cells. On the other hand, the regulatory population exerts a positive effect on Teff migration, anergy and death (*γ_E_*). That is, the presence of Treg cells triggers specific mechanisms which produces an increase of the apoptosis and anergic rate of activated Teff population, like its migration to the tissue [28,51]. This flow of Teff (*γ_E_*) controls the permanence expectancy of Teff in the activated proliferative state. Since this expectancy of the Teff population depends on the Treg density at each moment; it increases to 5 days in the absence of activated Treg cells. This maximum expectancy (5 days) was estimated based on the knowledge that the activated-T cells migrate to the target tissue 5-7 days after activation [51]. The estimated value for the permanence expectancy of the activated-Treg cells in the draining lymph node (*γ_R_*) is independent of the Teff population in our model and keeps constant at 5 days. In summary, the negative influence of Treg on activated-Teff cells resides in the inhibitory signals produced by activated-Treg cells, which impair their proliferation [17,52] and promote both apoptosis [50] and migration to the tissue [28].

There is experimental evidence of the existence of a positive effect of Teff cells over Treg population: Treg cells proliferate in response to immunization in vivo [28,40,53], and in vitro when stimulated with a TCR trigger together with Teff cells (28). This contrast with the previous notion than Treg were anergic, but the lack of proliferation of Treg in early experiments were due to the in vitro conditions and the new in vivo data demonstrate they can expand after immune system activation [54]. They proliferate and accumulate locally in response to transgenically expressed tissue antigen whereas their CD25^- ^cell counterparts are depleted at such sites [54]. These observations support a model in which Treg population dynamics are shaped by the local environment. Therefore, there is a positive influence of Teff cells over Treg population: Activated Teff cells produce signals that evoke the expansion of activated Treg cells and that enhance their suppressive function in vitro and in vivo [29]. The positive influence was also assumed to operate according to a Hill function which links the activation of Teff cells with the expansion and regulatory activity of Treg cells [29]. Thus the suppressor activity of Treg become stronger in the presence of increasing levels of Teff cells, mimicking molecular mechanisms such as conversion of Teff to the Tr1 phenotype after stimulation [24,52], or the influence of IL-2 [40,55,56].

#### T-cell activation

Resting auto-reactive Treg and Teff cells are unable to participate in an immune response until they become activated by antigen on APCs, including self-antigens [39], through molecular mimicry between self-antigens and other antigens, or other proposed mechanisms [47]. Since we aim to study the regulation between activated T populations, T-cell activation by APC was modelled as a deterministic process for simplicity: every resting T cell which is produced by the thymus will become activated after 24 hours, since T-cell activation by APC extends from 16 to 28 hours [57]. Once cells are activated, the cross-regulation between activated Teff and Treg cells defining the Teff-Treg loop modulates the outcome of the immune response.

#### Stochastic process

Stochastic fluctuations in biological systems are ubiquitous and may drastically modify the deterministic predictions [41,58,59]. Gaussian white noise variations are usually added to the predator death rate and prey birth rate to model the continuous environmental fluctuations [60]. However, to gain more realistic and general understanding of the effect of environmental fluctuations leading to extinction of the species, it is highly desirable to adopt so-called stochastic pulse trains rather than Gaussian white noise as the description of fluctuations [41]. The thymic production of new resting T cells is essentially a stochastic process with a series of discrete events as cells with a relevant specificity happen to be produced by V(D)J recombination and exported after maturation. In this way we modelled the influx of resting cells as spike trains instead of Gaussian noise. In this case it is desirable to take into account discrete and drastic actions for modelling the appearance of new resting/naïve T cells, which are going to be activated. We did not use a Gaussian white noise since it always assumes the presence of continuous perturbations, while in real systems there are some unavoidable sparse, yet drastic, impulses that may qualitatively change the system behaviour and even completely invalidate the deterministic predictions. As a consequence, the stochastic perturbations are intrinsically non-Gaussian and a discrete fluctuation model was preferred. In the present model, the escape of self-reactive naïve T-cells from deletion in the thymus and self-reactive resting T-cells which are capable to be activated under specific environmental triggers, such as infections, were modelled using a stochastic function by uniformly distributed production of resting Teff and Treg cells over time. The Teff and Treg perturbations represent trains of 100 randomly distributed impulses per year. This modelling reflected the fact that the generation of a given self-reacting T-cell is a random event and that consecutive events are not related to one another [61]. Changing the seed of the random number generator corresponds to the generation of T-cells at different times along the course of the simulation, but maintaining the same frequency and distribution of cell production. Due to these random perturbations, the model becomes a system of stochastic differential equations where the Teff-Treg loop is fed with a small noise input of new resting T cells.

### Simulations

Simulations are initiated with 0 resting Teff and Treg cells, and 1,000 and 200 activated Teff and Treg cells respectively. Reversible and irreversible damage levels are initiated with 0. We performed a sensitivity analysis of wide range of values the different proliferation rates (*α_E_*) and (*α_R_*). The time span of each simulation was 1,825 days (5 years). This period was chosen because clinical relapses in autoimmune diseases such as MS range from months to years and the simulations were intended to represent this process. Depending on the values of *α_E _*and *α_R_*, the evolution of activated-Teff cells may differ. Simulations of the healthy regime (tolerance, immune homeostasis) were carried out using *α_E _= 2 *and *α_R _= 1*. For the autoimmune regime we used the *α_R _= 0.25 *parameter, which reflected a weak response of the Treg population in autoimmune diseases. The amplitude of Teff cell burst was similar to experimental data [28].

### Sensitivity analysis

Sensitivity analysis of the T-cell population model was performed by running simulations over 1,825 days for each pair of proliferation rate values in the range of *α_E _= *[1:2] and *α_R _= *[0.25: 1], with a time resolution of 0.05 days (the parameter values were tested in increments of 0.05). The maximum value reached by the activated-Te cells was retained from each simulation. Since we are dealing with stochastic differential equations and we are interested in extracting the global behavior of our system, the procedure was repeated for a large number of seeds (200 different realizations of the noise for each pair of values of the scanned parameters). We checked that 200 simulations per each pair of proliferation rates were enough to reach reasonably convergent results (the convergence for the frequency is much faster than the convergence for the intensity of the relapses). We empirically calculated the convergence when the difference between the maximum and minimum value of the signal is less than 10^-5^.

### Correlation analysis between simulations and clinical time series

In an attempt to compare the clinical data from MRI (CEL series) and the simulated series of the reversible tissue damage, we made two simple processing steps. First, in order to have the same temporal resolution in both series (real and simulated data), the simulated series were discretized by taking only one point per month. Second, because the experimental and theoretical observables are not directly comparable, we calculated the increment or decrement of the variable in both time series types at each time t. This was done by subtracting the value at t-1 from the value at t for the whole series. Hence, new processed series have a value of zero when the disease neither deteriorates nor improves. Positive values represent the disease exacerbation and negative values a decrease in the disease activity. For clarifying, we denominated the processed series from real data (CEL time series) and from the simulations as "disease evolution" and "in-silico evolution" respectively. We calculated the "disease evolution" for the nine patients with MS whom underwent monthly MRI for 48 months. The "in-silico evolution" was also calculated for 2000 simulations under autoimmune configuration (α_R _= 0.25) with a time window of 120 months. That is, the "in-silico evolution series" are longer than the 9 "disease evolution series". The best Pearson's correlation coefficient when sliding each "disease evolution series" (48 points) along the "in-silico evolution series" (120 points) was obtained and plotted in a histogram (Figure 5).

## Abbreviations

Teff: effector T cell; Treg: regulatory T cell; APC: Antigen presenting cells; MS: Multiple Sclerosis; CNS: Central Nervous System.

## Authors' contributions

NV created the model, performed the analysis and wrote the paper; JC supervised the model and parameter estimation; RS participated in the original idea and performed the comparison between time series and model simulations; JG contributed to model development and software implementation; JB contributed to the time series analysis; SMP, IMF and JS contributed to parameter estimation by searching the biological literature; JT contributed to model and software implementation; FB generated MRI data and collected clinical information from patients with MS at the NIH; JGO supervised the model and its analysis; PV designed the study, supervised the model implementation and analysis and wrote the paper. All authors read and approved the final manuscript.

## Supplementary Material

Additional file 1 **Model equations for Vensim software as text**. It contains all the equations, auxiliary variable definitions and parameter values of the model (doc file). Click here for file

Additional file 2 **The model in Vensim software code**. It contains the model. It's ready to run using the Vensim software (mdl file). Vensim can be downloaded from http://www.vensim.com/ Click here for file

Additional file 3 **Dataset CEL from patients with MS Data from 9 patients with RRMS who underwent monthly MRI with gadolinium for 48 months**. It showed the number of CELs for each consecutive month on the MRI, the Expanded Disability Status Scale (EDSS) for measuring clinical disability and the presence of clinical relapses (excel file). Click here for file

Additional file 4 **Figure S1. Clinical heterogeneity: Influence of timing on the generation of self-reacting T-cells in the dynamics of the immune system**. Four simulations (over 5 years) of the same model (same parameters and initial conditions) are presented for four different seeds generating the naïve Teff and Treg populations. We used a parameter configuration that allows the generation of autoimmune dynamics (α_R _= 0.25). Left: Dynamics of activated Teff cells. Right: Evolution along time of the reversible, irreversible and total damage. Click here for file

Additional file 5 **Figure S2**. Sensitivity analysis of the model A. At individual level: The figure shows the sensitivity analysis when changing the Teff and Treg proliferation rates for four different seeds. The X axis corresponds to the maximum Treg proliferation rate, α_R_, and the Y axis to the maximum Teff proliferation rate α_E_. The Z axis shows the average relapse intensity reached by activated-Teff cells during a 5 year simulation (Z axis). Each blanket corresponds to a different seed. B. At global level: We realize 200 different simulations per each pair of proliferation rates; Average of 200 different "A" blankets. Click here for file

Additional file 6 **Figure S3**. The effect of perturbing the Te-Treg loop with a pulse of Treg and Te cells. The system, under autoimmune configuration, was perturbed at different times (sectors) with different intensity pulses of Treg/Te cells and the graphs display the phase space of the Te-Tr loop. The number of activated-Treg cells is plotted on the *X *axis and the number of the activated-Te cells on the *Y *axis. Both populations move clockwise along a spiral path to the equilibrium point in the absence of perturbations. Because the Te and Treg populations fluctuate under a negative feedback control, there are four feasible dynamic states. Sector I: both activated Te and Treg populations are growing. Sector II: the activated-Treg population is growing and the activated-Te population is diminishing. Sector III: the activated-Treg population is diminishing and activated-Te population is growing. Sector IV: the activated-Te population is growing and activated-Tr population is diminishing. The trajectory before the perturbation is depicted in black and after in red. Treg impulses: A. A small Treg perturbation in sector I leads to a jump to a closer trajectory. B. A large Treg perturbation in sector I leads to a jump to a more distant trajectory. C. a small Treg perturbation in sector IV leads to a jump to a closer trajectory. D. a large Treg perturbation in sector IV leads to a jump to a more distant trajectory. Any other perturbation in sector II and III, irrespective of its intensity, will move the system to another more distant trajectory (data not shown). Te impulses: E. A small Te perturbation in sector IV leads to a jump to a closer trajectory. F. a large Te perturbation in sector IV leads to a jump to a more distant trajectory. G. A small Te perturbation in sector III leads to a jump to a closer trajectory. H. A large Te perturbation in sector III leads to a jump to a more distant trajectory. Any other perturbation in sector I and II will move the system to a more distant trajectory, irrespective of its intensity (data not shown). Click here for file
