# Supplementary material for: Modeling the effector - regulatory T cell cross-regulation reveals the intrinsic character of relapses in Multiple Sclerosis
Source: BMC Syst Biol. 2011 Jul 15;5:114. doi: 10.1186/1752-0509-5-114 (PMC3155504; doi:10.1186/1752-0509-5-114)
Supplement: Additional file 1 — Model equations for Vensim software as text. It contains all the equations, auxiliary variable definitions and parameter values of the model (doc file). [file 1752-0509-5-114-S1.DOC]

### Model equations for Vensim software

# Levels

Resting Teff= INTEG (Naïve Teff Input - Teff Activation – Teff Anergy, 0)

Resting Treg = INTEG (Naïve Treg Input - Treg Activation – Treg Anergy, 0)

Activated Teff = INTEG (+Teff Activation – Teff Memory - Teff Death Deactivation Migration + Teff Proliferation, 1000)

Activated Treg = INTEG (Treg Activation + Treg Proliferation - Treg Death Deactivation Migration, 200)

# Flows

Naïve Teff Input = IF THEN ELSE(RANDOM UNIFORM(0, 1, NOISE SEED) < (100 * TIME STEP / 365), 100 / TIME STEP, 0)

Naïve Treg Input = IF THEN ELSE(RANDOM UNIFORM(0, 1, NOISE SEED) < (100 * TIME STEP/365), 100 / TIME STEP, 0)

Teff Activation = Antigen Presentation * Naïve Teff

Treg Activation = Naïve Treg * Antigen Presentation

Teff Memory = Activated Teff * gamma

Treg Memory = Activated Treg * gamma

Teff Anergy= Resting Teff * gamma

Treg Anergy = Resting Treg * gamma

Teff Proliferation = Activated Teff * Teff Proliferation Rate

Teff Death Deactivation Migration = Activated Teff / Teff Death or Deactivation Time

Treg Activation and Proliferation = Activated Treg * Treg activation and Proliferation Rate

Treg Death Deactivation Migration = Activated Treg / Treg Death or Deactivation Time

# Auxiliary equations

Activation by Teeg of Regulatory Mechanisms=(Activated Te^h/(ke^h+Activated Teff^h))

Activation by Treg of Citotoxic Mechanisms=kr^h/(kr^h+Activated Treg^h)

Activation by Treg of Inhibitory Mechanisms=kr^h/(kr^h+Activated Treg^h)

Teff Proliferation Rate = Max Te Proliferation Rate*Activation by Tr of Inhibitory Mechanisms

Teff Death Deactivation Migration Time=(Activation by Tr of Citotoxic Mechanisms*Max Te Death Deactivation Migration Time)+1

Treg activation and Proliferation Rate = Activation by Teff of Regulatory Mechanisms * Max Treg activation and Proliferation Rate

**Constants**

AntigenPresentation = 24

Ke = 1000

Kr = 200

h=5

Max Teff Proliferation Rate = 2 %[1:0.05:2]

Max Treg Proliferation Rate = 0.25 %[0.25:0.05:1]

NOISE SEED = 1 %[1:1:200]

Max Teff Death or Deactivation Time = 5

Treg Death or Deactivation Time = 5

Gamma = 0.05

# Simulation parameters

INITIAL TIME = 0 (Units: Day)

FINAL TIME = 1825 (Units: Day)

TIME STEP = 0.01

SAVEPER = 0.01 (Save results every TIME STEP)
