# Supplementary material for: Modeling the effector - regulatory T cell cross-regulation reveals the intrinsic character of relapses in Multiple Sclerosis
Source: BMC Syst Biol. 2011 Jul 15;5:114. doi: 10.1186/1752-0509-5-114 (PMC3155504; doi:10.1186/1752-0509-5-114)

**Figure S1. Clinical heterogeneity: Influence of timing on the generation of self-reacting T-cells in the dynamics of the immune system**. Four simulations (over 5 years) of the same model (same parameters and initial conditions) are presented for four different seeds generating the naïve Teff and Treg populations. We used a parameter configuration that allows the generation of autoimmune dynamics ($\alpha_{R}=0.25$). Left: Dynamics of activated Teff cells. Right: Evolution along time of the reversible, irreversible and total damage.


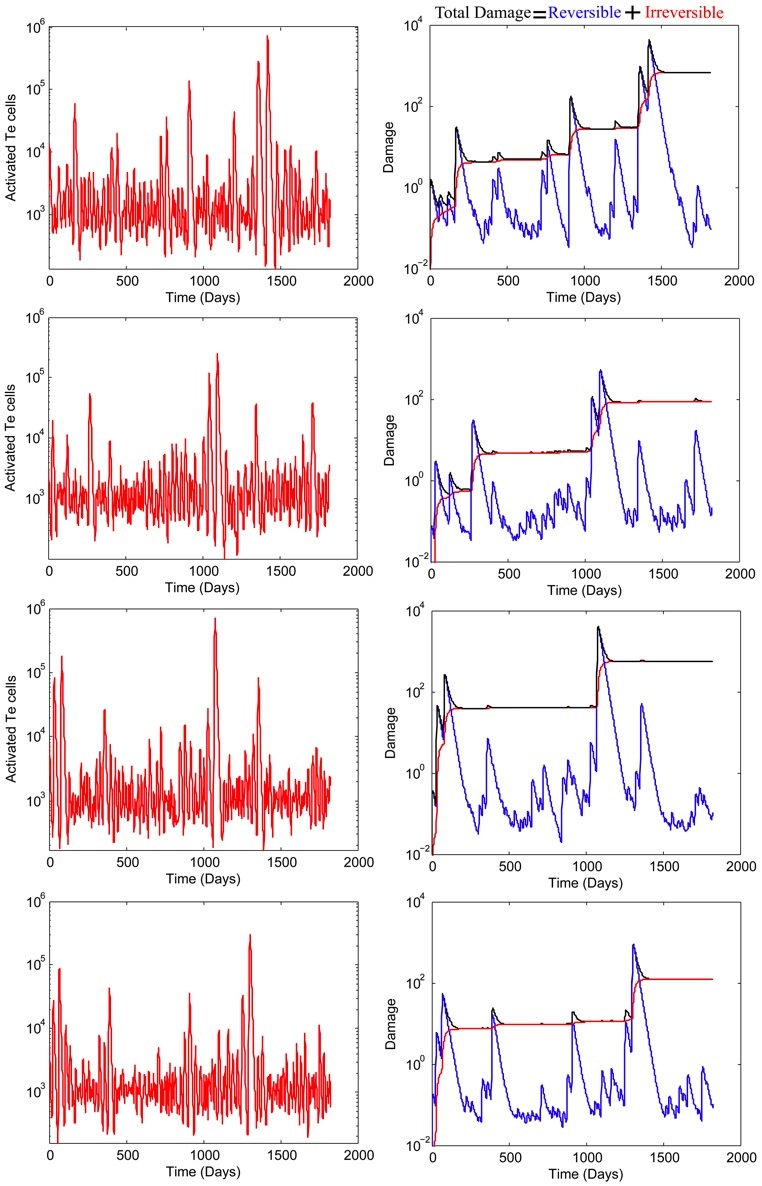

Supplement: Additional file 4 — Figure S1. Clinical heterogeneity: Influence of timing on the generation of self-reacting T-cells in the dynamics of the immune system. Four simulations (over 5 years) of the same model (same parameters and initial conditions) are presented for four different seeds generating the naïve Teff and Treg populations. We used a parameter configuration that allows the generation of autoimmune dynamics (αR = 0.25). Left: Dynamics of activated Teff cells. Right: Evolution along time of the reversible, irreversible and total damage. [file 1752-0509-5-114-S4.DOC]
