# Supplementary material for: Modeling the effector - regulatory T cell cross-regulation reveals the intrinsic character of relapses in Multiple Sclerosis
Source: BMC Syst Biol. 2011 Jul 15;5:114. doi: 10.1186/1752-0509-5-114 (PMC3155504; doi:10.1186/1752-0509-5-114)
Supplement: Additional file 5 — Figure S2. Sensitivity analysis of the model A. At individual level: The figure shows the sensitivity analysis when changing the Teff and Treg proliferation rates for four different seeds. The X axis corresponds to the maximum Treg proliferation rate, αR, and the Y axis to the maximum Teff proliferation rate αE. The Z axis shows the average relapse intensity reached by activated-Teff cells during a 5 year simulation (Z axis). Each blanket corresponds to a different seed. B. At global level: We realize 200 different simulations per each pair of proliferation rates; Average of 200 different "A" blankets. [file 1752-0509-5-114-S5.DOC]

**Figure S2. Sensitivity analysis of the model**. **A)** At individual level: The figure shows the sensitivity analysis when changing the Teff and Treg proliferation rates for four different seeds. The X axis corresponds to the maximum Treg proliferation rate, $\alpha_{R}$, and the Y axis to the maximum Teff proliferation rate $\alpha_{E}$. The Z axis shows the average relapse intensity reached by activated-Teff cells during a 5 year simulation (Z axis). Each blanket corresponds to a different seed. **B:** At global level: We realize 200 different simulations per each pair of proliferation rates; Average of 200 different “A” blankets.

**
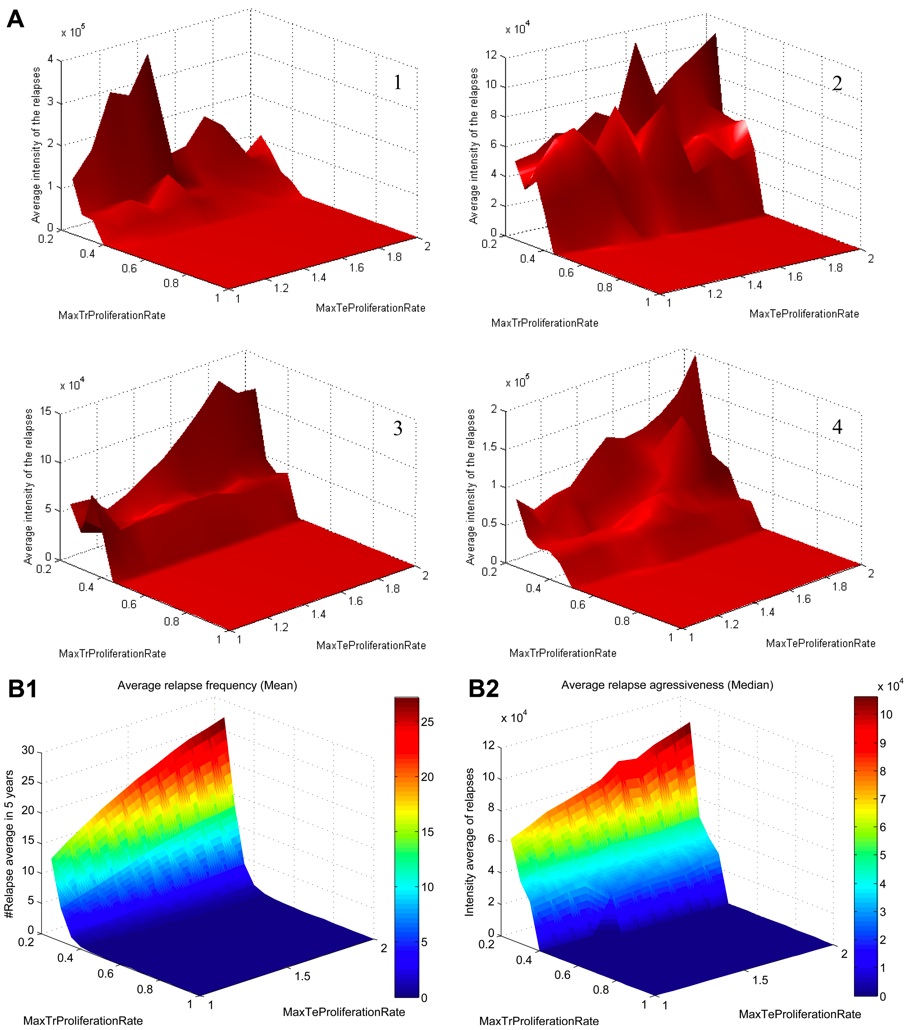
**
