# Supplementary material for: Modeling the effector - regulatory T cell cross-regulation reveals the intrinsic character of relapses in Multiple Sclerosis
Source: BMC Syst Biol. 2011 Jul 15;5:114. doi: 10.1186/1752-0509-5-114 (PMC3155504; doi:10.1186/1752-0509-5-114)
Supplement: Additional file 6 — Figure S3. The effect of perturbing the Te-Treg loop with a pulse of Treg and Te cells. The system, under autoimmune configuration, was perturbed at different times (sectors) with different intensity pulses of Treg/Te cells and the graphs display the phase space of the Te-Tr loop. The number of activated-Treg cells is plotted on the X axis and the number of the activated-Te cells on the Y axis. Both populations move clockwise along a spiral path to the equilibrium point in the absence of perturbations. Because the Te and Treg populations fluctuate under a negative feedback control, there are four feasible dynamic states. Sector I: both activated Te and Treg populations are growing. Sector II: the activated-Treg population is growing and the activated-Te population is diminishing. Sector III: the activated-Treg population is diminishing and activated-Te population is growing. Sector IV: the activated-Te population is growing and activated-Tr population is diminishing. The trajectory before the perturbation is depicted in black and after in red. Treg impulses: A. A small Treg perturbation in sector I leads to a jump to a closer trajectory. B. A large Treg perturbation in sector I leads to a jump to a more distant trajectory. C. a small Treg perturbation in sector IV leads to a jump to a closer trajectory. D. a large Treg perturbation in sector IV leads to a jump to a more distant trajectory. Any other perturbation in sector II and III, irrespective of its intensity, will move the system to another more distant trajectory (data not shown). Te impulses: E. A small Te perturbation in sector IV leads to a jump to a closer trajectory. F. a large Te perturbation in sector IV leads to a jump to a more distant trajectory. G. A small Te perturbation in sector III leads to a jump to a closer trajectory. H. A large Te perturbation in sector III leads to a jump to a more distant trajectory. Any other perturbation in sector I and II will move the system to a more distant trajector [file 1752-0509-5-114-S6.DOC]

**Figure S3. The effect of perturbing the Te-Treg loop with a pulse of Treg and Te cells**. The system, under autoimmune configuration, was perturbed at different times (sectors) with different intensity pulses of Treg/Te cells and the graphs display the phase space of the Te-Tr loop. **The number of activated-Treg cells is plotted on the *X* axis and the number of the activated-Te cells on the *Y* axis**. Both populations move clockwise along a spiral path to the equilibrium point in the absence of perturbations. Because the Te and Treg populations fluctuate under a negative feedback control, there are four feasible dynamic states. Sector I: both activated Te and Treg populations are growing. Sector II: the activated-Treg population is growing and the activated-Te population is diminishing. Sector III: the activated-Treg population is diminishing and activated-Te population is growing. Sector IV: the activated-Te population is growing and activated-Tr population is diminishing. The trajectory before the perturbation is depicted in black and after in red. Treg impulses: A. A small Treg perturbation in sector I leads to a jump to a closer trajectory. B. A large Treg perturbation in sector I leads to a jump to a more distant trajectory. C. a small Treg perturbation in sector IV leads to a jump to a closer trajectory. D. a large Treg perturbation in sector IV leads to a jump to a more distant trajectory. Any other perturbation in sector II and III, irrespective of its intensity, will move the system to another more distant trajectory (data not shown). Te impulses: E. A small Te perturbation in sector IV leads to a jump to a closer trajectory. F. a large Te perturbation in sector IV leads to a jump to a more distant trajectory. G. A small Te perturbation in sector III leads to a jump to a closer trajectory. H. A large Te perturbation in sector III leads to a jump to a more distant trajectory. Any other perturbation in sector I and II will move the system to a more distant trajectory, irrespective of its intensity (data not shown).

| **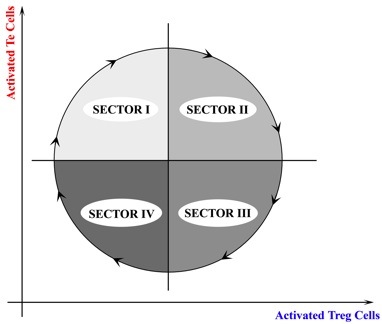** | |
| --- | --- |
| **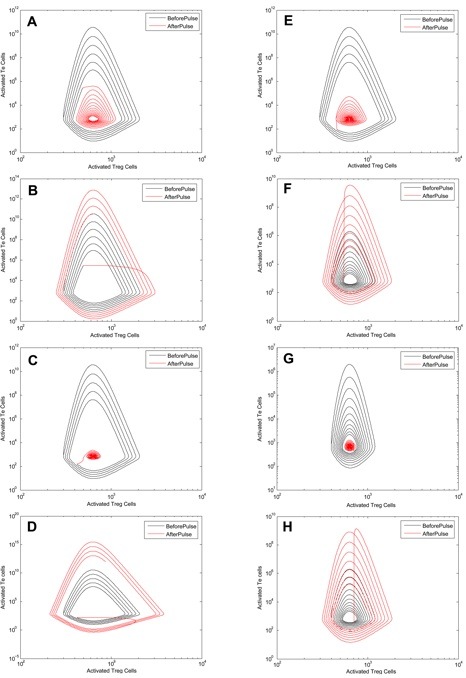** | **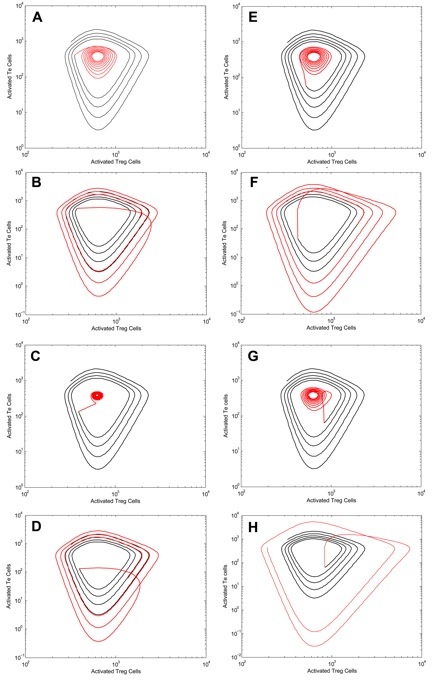** |
